# Hearing laughter improves the recovery process of the autonomic nervous system after a stress-loading task: a randomized controlled trial

**DOI:** 10.1186/s13030-018-0141-0

**Published:** 2018-12-21

**Authors:** Yoshiyasu Fujiwara, Hitoshi Okamura

**Affiliations:** 1 Graduate School of Health Sciences, Hiroshima University, Hiroshima, Japan; 2 School of Medicine, Nursing, Kurume University, 777-1 Higashi-kushihara-machi, Kurume-shi, Fukuoka 830-0003 Japan; 3 Graduate School of Biomedical & Health Sciences, Hiroshima University, 1-2-3 Kasumi, Minami-ku, Hiroshima 734-8551 Japan

**Keywords:** Stress, Relaxation, Laughter, Heart rate variability, Inter-beat intervals

## Abstract

**Background:**

It has become necessary to develop mental health management methods that do not require specialized skills or tools to implement. With this in mind, we performed a subjective and objective investigation of the stress-reducing effect of hearing laughter.

**Methods:**

Ninety healthy students were randomly assigned to a laughter (*n* = 45) or rest (*n* = 45) group. Both groups were then administered the Uchida-Kraepelin test for 15 min, which served as a stress-loading method. The laughter group listened to a specially prepared CD for five minutes, while the rest group rested for five minutes. The participants’ subjective stress level was assessed using a visual analogue scale and their physiological status was assessed by measuring blood pressure and monitoring heart rate variability.

**Results:**

The visual analogue scale score for subjective stress was found to decrease significantly in both the laughter and rest groups after the intervention. However, a two-way repeated-measures analysis of variance revealed significant interaction and main effects for the change in heart rate and the natural logarithm of the high-frequency component of heart rate variability (lnHF). A post-hoc analysis using Dunnett’s test showed that hearing laughter caused the lnHF to significantly increase compared to that recorded during the Uchida-Kraepelin test and the rest period.

**Conclusions:**

These results suggest that hearing laughter might produce a relaxing effect by increasing parasympathetic nervous activity. This would make it an easily accessible method for improving the recovery process of the autonomic nervous system after a stress-loading task that does not require specialized skills or tools.

**Trial registration:**

UMIN000016422. Retrospectively registered on 2 February 2015.

**Electronic supplementary material:**

The online version of this article (10.1186/s13030-018-0141-0) contains supplementary material, which is available to authorized users.

## Background

Approximately 121 million people worldwide are estimated to suffer from depression, and occupational stress is one of the main causes of this condition [1]. It has been reported that occupational stress not only affects individuals’ mental state, but also increases the risk of cardiovascular disease [2] and hypertension [3]; thus, appropriate mental health measures are urgently needed. A common measure currently used to counter occupational stress is improving the work environment [4, 5]. However, for some employees, this approach has returned unfavorable results. Other methods used for mental health management include cognitive-behavioral therapy [6], educational methods [7], and Tai Chi exercise interventions [8]. While these methods have been shown to be effective, their implementation requires specialized knowledge and skills.

There is, as such, a clear need for mental health management methods that do not require specialized skills or tools for their implementation; that is, methods that enable primary prevention and that can be implemented at a low cost without experts. Laughing has attracted attention as one such management method, and has previously been reported to have a stress-reducing effect [9, 10]. Encouragement of laughing behavior is advantageous—it can be routinely engaged in and is completely free of charge [11]. In fact, while personal relations in the workplace have been reported to increase the incidence of psychiatric disorders [12], it has been suggested that encouraging laughing may be an effective means of reducing such stress, as it can contribute to maintaining and improving personal relations in the workplace. Studies on laughter have, however, reported that laughing can be divided into several categories (e.g., Duchenne laughter, non-Duchenne laughter, requested laughter, tickling laughter) [13, 14] and is difficult to define as a specific activity. In addition, individuals’ subjectivity may influence the effect of laughing, even when individuals are participating in the same intervention. Further, it is also difficult to repeatedly perform such interventions under the same conditions. Therefore, in this study, we focused on “hearing laughter,” which is an aspect of laughing behavior. We hypothesized that hearing laughter might have a stress-reducing effect.

To objectively investigate the stress-reducing effect of hearing laughter, we decided to examine cardiac autonomic nervous activity. Many previous studies have investigated occupational stress from the standpoint of inter-beat intervals (IBIs), and found that stress inhibits cardiac vagal activity [15–17]. The method used in the present study consisted of an analysis of heart rate variability and IBIs using electrocardiography. Electrocardiography is a non-invasive measurement that causes little physical and psychological stress, and its ability to provide a continuous measurement would allow us to more smoothly monitor the changes between two time points (i.e., before and after the intervention).

To date, no studies have assessed the stress-reducing effect of hearing laughter, which occurs routinely and spontaneously in our daily lives. Considering this, the aim of the present study was to investigate the improvement of the recovery process of the autonomic nervous system after a stress-loading task, using not only a subjective measurement of stress but also by monitoring blood pressure, heart rate, and IBIs (i.e., cardiac autonomic nervous activity). If the effect of hearing laughter could be demonstrated in this study, it would facilitate the effective utilization of laughter as a mental health management tool.

## Methods

### Subject selection criteria

The subjects recruited for this study were healthy students attending a junior college of nursing in Japan. Recruitment information was posted on paper flyers around campus to solicit voluntary participation. In order to eliminate the possible influence of medicines and mental state, we chose to exclude menstruating women and individuals who were attending a hospital for treatment.

According to a power analysis performed using G*Power (version 3.1.9.2), the minimum required sample size of each group was 45. This is sufficient to obtain 80% statistical power at an alpha level of 0.05 with a large effect size (d = 0.60) between the laughter and rest groups.

### Experimental conditions

All aspects of the experiment, except for the interventions, were performed under the same conditions in a quiet room. The subjects were asked to avoid vigorous exercise and alcohol on the day before and on the day of the experiment, and were prohibited from smoking and taking caffeine on the day of the experiment. This limitations were put in place to reduce the risk of abnormal alterations to the autonomic nervous system. All subjects were requested to fast overnight, and were only allowed to consume water in the three hours before the experiment began. At the start of the experiment, the room had a mean temperature of 22.8 ± 1.2 °C, a mean humidity of 30.0% ± 5.2%, and a mean equivalent continuous noise level (LAeq) of 48.7 ± 2.9 db.

### Experimental procedures

Using the envelope method, eligible subjects were randomly assigned, at a 1:1 ratio, to a “hearing laughter” group (laughter group) or a rest group by the researcher in charge of the allocation. For both groups, the entire experiment was performed with the subjects at rest, sitting in a chair. After providing their basic information (age and sex), subjects were asked to wear the heartbeat sensor strap of the heart rate monitor around the underbust area, as well as a transmitter on their arm. They then rested for 10 min. Subsequently, their blood pressure was measured, and the subjects were asked to complete a visual analogue scale (VAS) measuring subjective stress. They then underwent the Uchida-Kraepelin test for 15 min, which served as a stress-loading exercise. After the Uchida-Kraepelin test, subjects’ blood pressure was measured again and they were again asked to complete the VAS.

Subsequently, subjects underwent the assigned intervention (laughter or rest) for five minutes: the laughter group listened to a CD comprising sounds of people laughing, while the rest group rested. The CD used in the laughter intervention contained a combination of laughter from one person and laughter from several people, in order to ensure that the situation closely resembled a natural incident in daily life. The voices used were selected from sound-effect CDs (Sound Effect Complete Collection 16®, Best Sound Effects®, New Sound Effect Complete Collection®, and New Sound Effect Complete Collection 43®; King Record Co.).

During the intervention, the subjects were asked to maintain a respiratory rate of 15 breaths/minute (once every four seconds); a metronome was used to help them in this regard. We employed controlled breathing to eliminate the influence that changes in respiratory rate could have on heart rate and IBIs. After both interventions, we measured participants’ blood pressure again and they completed the VAS a final time.

**Fig. 1:**
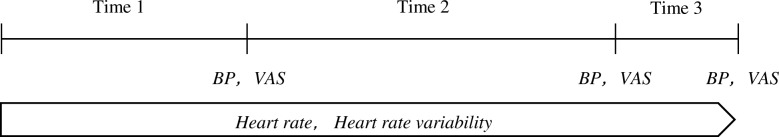
Flow of the experimental procedure. Time 1: During rest (10 min), Time 2: During the Uchida-Kraepelin test (15 min), Time 3: During each intervention (5 min). *BP*: blood pressure, *VAS*: visual analogue scale

Figure 1 depicts a simple diagram describing the flow of the experimental procedure.

### Measurements

#### Basic information

A self-administered questionnaire was used to obtain information on the age and sex of the subjects.

#### Subjective stress state

A VAS was used to measure participants’ subjective stress state. We presented the subjects with a 100-mm-long straight line, with the left and right ends corresponding to stress-free and maximum stress states, respectively, and asked them to rate their current stress levels by drawing a vertical line at the appropriate position on the line. The distance (mm) of the vertical line from the left end was measured for the assessment.

#### Blood pressure

Blood pressure was measured using an automatic digital sphygmomanometer (HEM-622, Omron Co.).

#### Heart rate variability

We measured IBIs using a heart rate monitor (model RS800CX, Polar Co.) designed to measure exercise capacity. A number of studies have used Polar heart rate monitors to analyze heart rate variability, and these studies have reported a good correlation between this system and the results of similar analyses based on electrocardiography [18–20]. Considering the temporary changes that inevitably occur immediately after the commencement of an experiment, we did not use any data obtained during the first minute of the experiment. We calculated the mean heart rate during the initial 10-min rest, during the 15-min stress-loading exercise, and during the 5-min intervention. We also performed a frequency analysis of the IBIs and calculated the very-low-frequency (VLF, < 0.04 Hz) component, low-frequency (LF, 0.04–0.15 Hz) component, and high-frequency (HF, 0.15–0.40 Hz) component. The LF component is said to reflect approximately 10-s arterial blood pressure oscillations triggered by arterial baroreceptors that are sensitive to fluctuations in arterial blood pressure (Mayer-wave-related sinus arrhythmia), while the HF component reflects breathing cycles triggered by pulmonary stretch receptors that are sensitive to the expansion and contraction of the lungs in response to breathing (respiratory sinus arrhythmia). In the past, the LF component has been used as an index of sympathetic nervous activity, the HF component as an index of parasympathetic nervous activity, and LF/HF as an index of the balance between these types of activity. However, it is now thought that a variety of elements, including both parasympathetic and sympathetic nervous activities, baroreceptor activity, and the endocrine system, can influence the LF component [21, 22]. Therefore, for the present study, we focused on the HF component as an index of parasympathetic nervous activity. As the measurement data for heart rate variability were non-normal, the obtained HF component was transformed to the natural logarithm (lnHF), which was used as an index of parasympathetic nervous activity.

### Analysis methods

Sex ratio was compared between the groups using the χ^2^ test and, after confirming the normality of the data, the participants’ ages and mean heart rates were compared using a *t*-test. Blood pressure, VAS, and mean heart rate before the intervention were compared between the groups using the Mann-Whitney U test or the *t*-test. We performed a two-way repeated-measures analysis of variance (ANOVA) using mean heart rate and lnHF as the dependent variables; this was to assess differences between the two groups regarding the changes in each of these variables during the resting state, during the Uchida-Kraepelin test, and during the intervention. Additionally, we performed a two-way repeated-measures ANOVA using blood pressure and VAS as the dependent variables to assess differences between the two groups regarding the changes in these variables immediately before and after the Uchida-Kraepelin test and after the intervention. Dunnett’s multiple comparisons were performed to examine the change in each variable for each group.

For all the tests, the *p* values were two-sided, and the significance level was set at *p* < 0.05. IBM SPSS Statistics 22 was used for all statistical analyses.

## Results

### Subjects participating in the study (Fig. 2)

Ninety students (79 women and 11 men) with a mean age of 20.5 ± 1.4 years provided consent to participate in this study. These 90 students were randomly assigned to the laughter group (39 women and 6 men) and the rest group (40 women and 5 men). There were no significant differences between the two groups in terms of sex ratio (*p* = 0.999) or age (*p* = 0.390). Since there were no dropouts from either of the groups, the data for all 90 subjects were included in the evaluations.

**Fig. 2:**
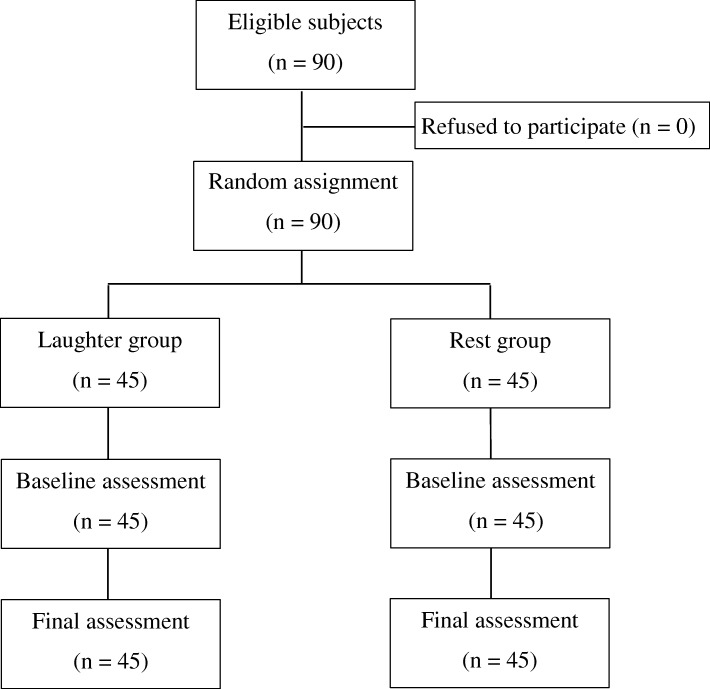
Flow of participants through the trial

### Comparison of blood pressure, VAS score, and mean heart rate before the intervention between the two groups

No significant differences in blood pressure, VAS score, or mean heart rate were observed between the two groups before the intervention (Table 1).

**Table 1:** Comparison of blood pressure, VAS score, and mean heart rate before the intervention between the two groups

	Laughter group (*n* = 45)	Rest group (*n* = 45)	*P* value
Systolic blood pressure			
Median (interquartile range)	103.00 (99.50–110.50)	104.00 (99.50–109.50)	0.961^a)^
Diastolic blood pressure			
Median (interquartile range)	67.00 (59.00–70.50)	67.00 (63.50–70.50)	0.403^a)^
VAS			
Median (interquartile range)	26.00 (8.50–54.50)	25.00 (7.00–41.50)	0.405^a)^
Mean Heart rate			
Mean (SD)	77.62 (7.78)	78.95 (7.69)	0.064^b)^

^a^: Mann-Whitney U test

^b^: *t*-test

*SD* standard deviation

*VAS* visual analogue scale

### Comparison of changes in mean heart rate, lnHF, blood pressure, and VAS score between the two groups

**Fig. 3:**
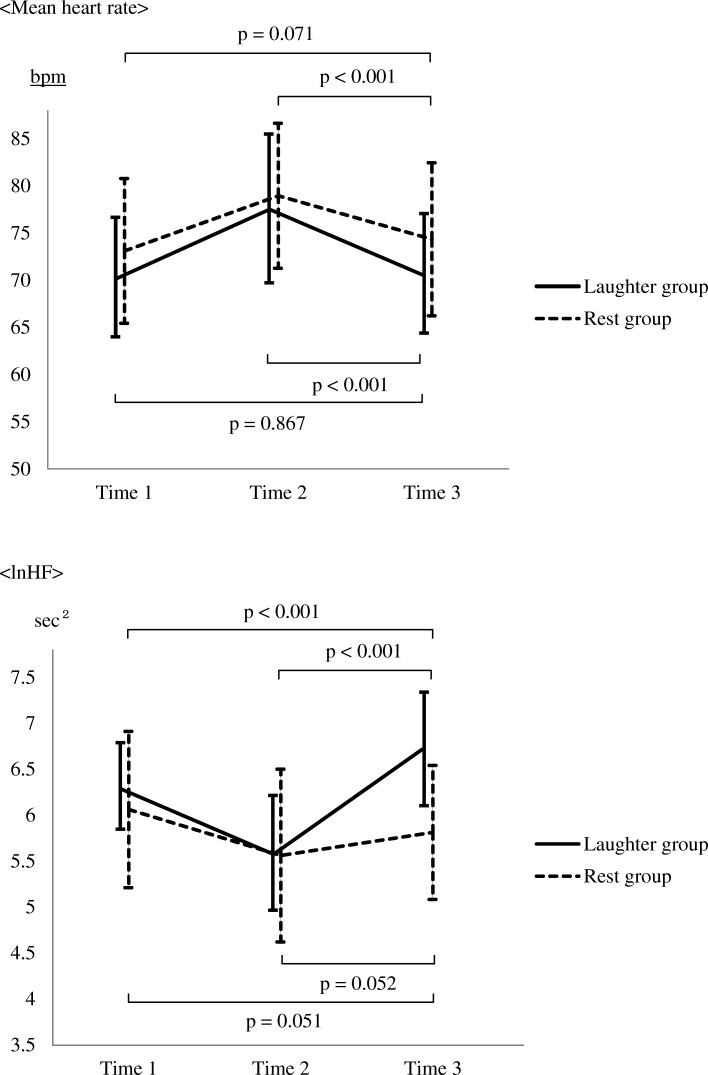
Changes between the two groups in mean heart rate and lnHF. Time 1: During rest (10 min), Time 2: During the Uchida-Kraepelin test. (15 min), Time 3: During each intervention (five minutes). Means are intersections between polygonal lines and vertical lines, and SDs are depicted as vertical lines

A two-way ANOVA revealed a significant interaction between the two groups for both the mean heart rate (*p* = 0.009) and the lnHF (*p* < 0.001). We also found a significant main effect of time for both the mean heart rate (*p* < 0.001) and the lnHF (*p* < 0.001), and a significant main effect of group for the lnHF (Table 2). For reference, we also show the changes in lnLF and lnLF/HF in each group before and after the intervention in a Additional file 1: Table S1. Multiple comparisons using Dunnett’s test revealed that in both groups (ps < 0.001) participants’ mean heart rate during the interventions significantly decreased compared to that during the Uchida-Kraepelin test. On the other hand, in the laughter group, the lnHF significantly increased during the intervention compared to that during the rest (*p* < 0.001) and that during the Uchida-Kraepelin test (*p* < 0.001). No significant change in the lnHF was observed in the rest group (Fig. 3).

**Table 2:** Changes in the mean heart rate and lnHF in each group before and after the intervention

	Laughter group (*n* = 45)	Rest Group (*n* = 45)	Interaction		Main effect			
			Group × Time		Time		Group	
	Mean (SD)		F	P	F	P	F	P
Mean heart rate								
Time 1	70.31 (6.32)	73.10 (7.68)	4.80	0.009	163.1	< 0.001	3.15	0.079
Time 2	77.62 (7.78)	78.95 (7.69)						
Time 3	70.55 (6.38)	74.34 (8.11)						
lnHF								
Time 1	6.31 (0.48)	6.06 (0.85)	23.9	< 0.001	63.6	< 0.001	8.62	0.004
Time 2	5.58 (0.63)	5.56 (0.94)						
Time 3	6.72 (0.62)	5.81 (0.73)						

Time 1: During rest (10 min)

Time 2: During the Uchida-Kraepelin test (15 min)

Time 3: During each intervention (five minutes)

*SD* standard deviation

*lnHF* natural logarithm of high-frequency component of heart rate variability

**Fig. 4:**
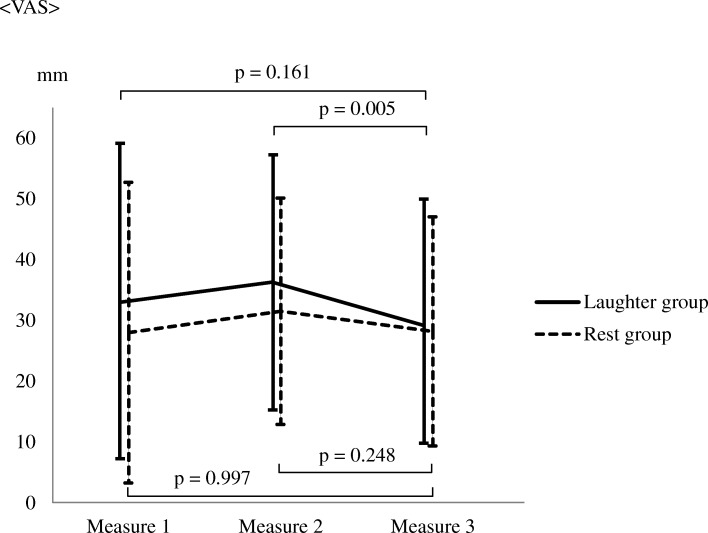
Changes between the two groups in terms of VAS. Measure 1: Before the Uchida-Kraepelin test, Measure 2: After the Uchida-Kraepelin Test, Measure 3: After the intervention. Means are intersections between polygonal lines and vertical lines, and SDs are depicted as vertical lines

Furthermore, no significant interaction effect of group and time was found for either blood pressure or VAS score. However, multiple comparisons revealed that only in the laughter group, the VAS score after the intervention significantly decreased compared to that before intervention (*p* = 0.005) (Table 3, Fig. 4).

**Table 3:** Changes in blood pressure and VAS in each group before and after the intervention

	Laughter group	Rest group	Interaction		Main effect			
	(*n* = 45)	(*n* = 45)	Group × Time		Time		Group	
	Mean (SD)		F	P	F	P	F	P
SBP								
Measure 1	105.40 (8.78)	105.13 (8.06)	0.74	0.477	6.54	0.002	0.45	0.503
Measure 2	108.71 (12.62)	107.86 (9.85)						
Measure 3	106.95 (9.34)	104.57 (7.85)						
DBP								
Measure 1	65.48 (7.63)	67.28 (8.37)	0.44	0.640	0.15	0.859	1.25	0.266
Measure 2	65.80 (7.39)	67.95 (8.45)						
Measure 3	66.37 (8.89)	66.93 (7.14)						
VAS								
Measure 1	32.97 (26.00)	28.06 (24.73)	0.96	0.383	5.37	0.005	0.68	0.412
Measure 2	36.13 (20.96)	31.55 (18.63)						
Measure 3	29.13 (20.34)	28.22 (18.83)						

Measure 1: Before the Uchida-Kraepelin test

Measure 2: After the Uchida-Kraepelin test

Measure 3: After the intervention

*SBP* Systolic blood pressure

*DBP* Diastolic blood pressure

*VAS* visual analogue scale

*SD* standard deviation

## Discussion

Psychological stress and elevated mood are associated with enhanced sympathetic nervous activity, while a relaxed state is associated with greater parasympathetic nervous activity. In this study, we assessed the changes in psychological stress levels using an objective indicator, cardiac autonomic nervous activity (in terms of heart rate variability and blood pressure). Furthermore, a VAS was used to assess subjective psychological stress levels.

There was no interaction between group and time for blood pressure. This is perhaps because the majority of the subjects were young healthy women, whose blood vessels are typically more elastic. As such, their blood pressure did not change to such an extent that an inter-group difference would be detectable in such a small sample.

Two-way repeated-measures ANOVAs showed significant interactions and main effects for the changes in mean heart rate and lnHF. A post-hoc analysis showed that while hearing laughter, subjects’ lnHF significantly increased compared to that during the rest and that during the Uchida-Kraepelin test. The results suggest that the recovery process of parasympathetic nervous activity, which was suppressed by the stress loading exercise, is enhanced by hearing laughter. A previous study on laughing showed that the HF component of heart rate variability decreased during laughing, but soon returned to the level before laughing [23]. A possible reason for the difference between this previous study and the present study is that listening to laughter is not accompanied by the physical activity of laughing. Law et al. [24] also reported that a simulated laugh led to an increase in heart rate and a decrease in parasympathetic nervous system activity, adding further evidence to the idea that the physical act of laughter underlies the differences between the studies.

Yasushi et al. [25] reported that HF component power and heart rate can vary depending on participants’ degree of drowsiness. They reported that, since sympathetic nervous activity changes from a stimulated to a suppressed state in situations in which subjects exhibit signs of drowsiness (i.e., when they were not aware of being drowsy, but have the physiological signs of drowsiness), the subjects exhibit a reduced heart rate, and since parasympathetic nervous activity is stimulated in situations in which an individual is drowsy, heart rate decreases and HF power increases. In the present study, we obtained similar results. Further, we also found that the VAS score, which was used to assess subjective psychological stress levels, significantly decreased after the intervention compared to before it in the laughter group. This finding suggests that hearing laughter causes a greater increase in parasympathetic nervous activity, which decreased during the stress loading, than does resting.

Heart rate variability is influenced by respiration, and the frequency band varies with respiratory rate, tidal volume, etc. [26]. However, the present study employed an intervention in which the subjects heard laughter while in a seated position at rest (i.e., in a physically inactive condition). In addition, the respiratory rate was controlled at 15 breaths/minute (once every four seconds), suggesting that the change in the HF was unlikely to have been caused by either physical activity or respiration.

This study had several limitations. First, the sample comprised mostly female students of around 20 years of age. However, individuals at greatest risk of high stress include patients with chronic medical disease or middle-aged or older male workers (who are at particular risk of cardiovascular events) exhibiting a lack of discretion or unfavorable interpersonal relationships at their workplace. Additionally, we expect that autonomic nervous responses to hearing laughter would be diminished in middle-aged or older male/female workers compared to those of young students. Therefore, our study cannot be directly linked to stress management in the occupational field, and is better situated as a preliminary laboratory experiment. Second, the subjects in the present study were young healthy college students whose stress was not so high. Therefore, we required a stress-loading task in order to induce a stress state. Third, “stress” has various meanings. However, in the present study, participants were asked only “How is your present stress level?” in the VAS; thus, their emotions/feelings were not sufficiently estimated. Fourth, we used only a single intervention trial. In the future, testing the effect of multiple trials is necessary, as it might result in greater attenuation of autonomic nervous responses.

In this study, we found that listening to laughter improved the recovery process of the autonomic nervous system after a stress load. Furthermore, significant improvements were observed in a VAS evaluating subjective psychological stress levels. Taken together, these results suggest that listening to laughter can bring about a stress alleviation effect. Hearing laughter does not depend on personal preferences, unlike music; is highly cost-effective in that it can be performed without special knowledge, skills or expense; and can be adopted in any work environment. In the future, it will be necessary to perform interventions and assessments in environments similar to those of actual workplaces.

## Additional file

Additional file 1: **Table S1.** Changes in lnLF and lnLF/HF in each group before and after the intervention. (DOCX 18 kb)

## Abbreviations

ANOVA: Analysis of variance
HF: High frequency
HRV: Heart rate variability
IBIs: Inter-beat intervals
LF: Low frequency
VAS: Visual analogue scale
VLF: Very low frequency
