# Supplementary material for: Hearing laughter improves the recovery process of the autonomic nervous system after a stress-loading task: a randomized controlled trial
Source: Biopsychosoc Med. 2018 Dec 21;12:22. doi: 10.1186/s13030-018-0141-0 (PMC6302464; doi:10.1186/s13030-018-0141-0)
Supplement: Supplementary file 1 — Table S1. Changes in lnLF and lnLF/HF in each group before and after the intervention. (DOCX 18 kb) [file 13030_2018_141_MOESM1_ESM.docx]

Additional file 1: Table S1. Changes in lnLF and lnLF/HF in each group before and after the intervention

|  | Laughter group  (n = 45) | Rest  Group  (n = 45) | Interaction | | Main effect | | | |
| --- | --- | --- | --- | --- | --- | --- | --- | --- |
|  |  |  | Group ×　time | | Time | | Group | |
|  | Mean (SD) | | F | P | F | P | F | P |
| lnLF |  |  |  |  |  |  |  |  |
| Time 1  Time 2  Time 3 | 6.86 (0.42)  6.28 (0.52)  6.91 (0.63) | 6.49 (0.59)  6.17 (0.58)  6.58 (0.66) | 3.36 | 0.037 | 51.3 | <0.001 | 6.88 | 0.01 |
| lnLF/HF  Time 1  Time 2  Time 3 | 0.49 (0.56)  0.69 (0.61)  0.30 (0.55) | 0.75 (0.66)  0.91 (0.73)  0.95 (0.73) | 6.09 | 0.003 | 4.21 | 0.016 | 11.8 | 0.01 |

Time 1: During rest (10 minutes)

Time 2: During the Uchida-Kraepelin test (15 minutes)

Time 3: During each intervention (five minutes)

SD: standard deviation

lnLF: natural logarithm of the low frequency component

lnLF/HF: natural logarithm of the high- and low-frequency ratio
